# Supplementary material for: Optimization of differentiation and transcriptomic profile of THP-1 cells into macrophage by PMA
Source: PLoS One. 2023 Jul 17;18(7):e0286056. doi: 10.1371/journal.pone.0286056 (PMC10351730; doi:10.1371/journal.pone.0286056)
Supplement: S1 File — (ZIP) [file pone.0286056.s001.zip › supporting files/RNA-seq report.zip!/RNA-seq report/5_KEGGEnrichment_result/induce_vs_control/Enrich_plot/induce_vs_control.DEG_up_KEGG_enriched.pdf]

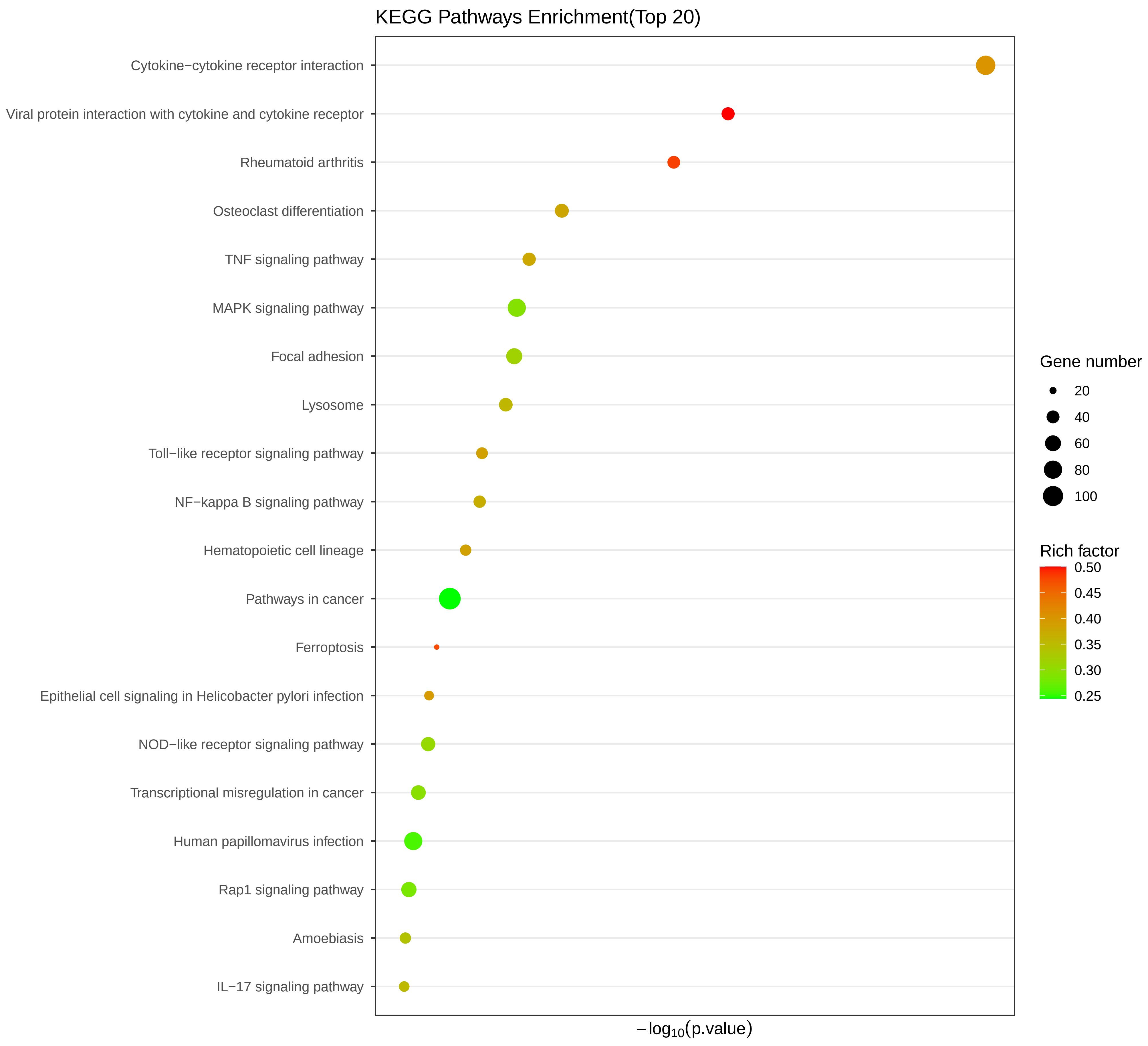
KEGG Pathways Enrichment(Top 20)
Cytokine−cytokine receptor interaction
Viral protein interaction with cytokine and cytokine receptor
Rheumatoid arthritis
Osteoclast differentiation
TNF signaling pathway
MAPK signaling pathway
Focal adhesion
Lysosome
Toll−like receptor signaling pathway
NF−kappa B signaling pathway
Hematopoietic cell lineage
Pathways in cancer
Ferroptosis
Epithelial cell signaling in Helicobacter pylori infection
NOD−like receptor signaling pathway
Transcriptional misregulation in cancer
Human papillomavirus infection
Rap1 signaling pathway
Amoebiasis
IL−17 signaling pathway
−log10(p.value)
Gene number
20
40
60
80
100
Rich factor
0.50
0.45
0.40
0.35
0.30
0.25
